# The Impact of an Ultra-Early Postoperative MRI on Treatment of Lower Grade Glioma

**DOI:** 10.3390/cancers13122914

**Published:** 2021-06-10

**Authors:** Andrej Pala, Gregor Durner, Michael Braun, Bernd Schmitz, Christian Rainer Wirtz, Jan Coburger

**Affiliations:** 1 Department of Neurosurgery, University of Ulm, 89312 Günzburg, Germany; gregor.durner@googlemail.com (G.D.); rainer.wirtz@uniklinik-ulm.de (C.R.W.); jan.coburger@uni-ulm.de (J.C.); 2 Department of Neuroradiology, University of Ulm, 89312 Günzburg, Germany; michael.braun@bkh-guenzburg.de (M.B.); bernd.schmitz@uniklinik-ulm.de (B.S.)

**Keywords:** lower grade glioma, intraoperative MRI, ultra-early postoperative MRI

## Abstract

**Simple Summary:**

The decision to provide adjuvant treatment in lower grade glioma (LGG) is often based on presence of residual tumor after surgery. Differentiating tumor remnants and surgically induced artifacts can be challenging. Postoperative MRI performed 24 or 48 h after the surgery overestimates residual tumor volume. MRI scan in the first hour after surgery (ultra-early), or an intraoperative MRI after final resection, correlated best with residual tumor at 3 months follow up.

**Abstract:**

The timing of MRI imaging after surgical resection may have an important role in assessing the extent of resection (EoR) and in determining further treatment. The aim of our study was to evaluate the time dependency of T2 and FLAIR changes after surgery for LGG. The Log-Glio database of patients treated at our hospital from 2016 to 2021 was searched for patients >18a and non-enhancing intra-axial lesion with complete MR-imaging protocol. A total of 16 patients matched the inclusion criteria and were thus selected for volumetric analysis. All patients received an intraoperative scan (iMRI) after complete tumor removal, an ultra-early postoperative scan after skin closure, an early MRI within 48 h and a late follow up MRI after 3–4 mo. Detailed volumetric analysis of FLAIR and T2 abnormalities was conducted. Demographic data and basic characteristics were also analyzed. An ultra-early postoperative MRI was performed within a median time of 30 min after skin closure and showed significantly lower FLAIR (*p* = 0.003) and T2 (*p* = 0.003) abnormalities when compared to early postoperative MRI (median 23.5 h), though no significant difference was found between ultra-early and late postoperative FLAIR (*p* = 0.422) and T2 (*p* = 0.575) images. A significant difference was calculated between early and late postoperative FLAIR (*p* = 0.005) and T2 (*p* = 0.019) MRI scans. Additionally, we found no significant difference between intraoperative and ultra-early FLAIR/T2 (*p* = 0.919 and 0.499), but we found a significant difference between iMRI and early MRI FLAIR/T2 (*p* = 0.027 and *p* = 0.035). Therefore, a postoperative MRI performed 24 h or 48 h might lead to false positive findings. An MRI scan in the first hour after surgery (ultra-early) correlated best with residual tumor at 3 months follow up. An iMRI with open skull, at the end of resection, was similar to an ultra-early MRI with regard to residual tumor.

## 1. Introduction

Lower grade gliomas (LGG) are rare and slow-growing infiltrative lesions typical in younger patients exhibiting mild symptoms [1,2]. Furthermore, the LGG is recurring and undergoes malignant transformation [3,4,5]. According to RTOG 9802 criteria, patients older than 40 years and/or less than gross total resection were assigned as high-risk patients typically resulting in adjuvant therapy [6]. Induced surgical artifacts on postoperative MRI scans may mimic residual tumors and therefore influence further therapy [7]. Based on our retrospective analysis, we proposed an ultra-early postoperative MRI directly after skin closure, or an intraoperative MRI without additional resection, as suitable imaging modalities for the further planning of postoperative therapy [8]. Since 2016, as part of the LogGlio registry for lower grade gliomas, all patients received imaging based on a standardized MRI protocol for preoperative, intraoperative and early and late postoperative imaging [9]. We have performed a detailed volumetric analysis of FLAIR/T2 changes using standardized MRI images of patients in the LogGlio study recruited by our department.

## 2. Materials and Methods

### 2.1. Patients and Follow-Up Assessmen

Patients included in the study were prospectively selected from the Log-Glio register of patients. It included those with a suspected diagnosis of LGG, based upon MRI scans. All patients selected were over the age of 18 and had signed informed consent between 2016 and 2020. The detailed study protocol is described in our earlier publication [9]. Basic tumor and patient characteristic included in the analysis were: age, gender, tumor location, IDH mutation, MGMT and 1p19q codeletion. Gliomas with contras enhancement were not included in the study.

### 2.2. OR Setup and MRI

An intraoperative 1.5 T MRI Espree scanner has been available (Espree, Siemens AG, Erlangen, Germany) at our department as a one-room solution since October 2008. During surgery, an intraoperative MRI scan was performed according to the surgeon’s decision. A ultra-early MRI was performed directly after skin closure with an intraoperative MRI. An early postoperative MRI was performed within 48 h using 1.5 or 3 T MRI after surgery and a late postoperative MRI was obtained 3–4 months after surgery using also 1.5 or 3 T scanner. The following sequences were performed:

1. Before surgery: T1 MPRAGE +/− Gadolinium enhancement, T2 SPACE, FLAIR 3D, DWI, PWI;
2. Intraoperative MRI: T1 MPRAGE, T2 SPACE, FLAIR 3D, DWI, (+/− Gadolinium enhancement, PWI);
3. Intraoperative after additional resection: T1 MPRAGE+/− Gadolinium enhancement, T2 SPACE, FLAIR 3D, DWI, PWI.

Postoperative within 48 h: T1 MPRAGE +/− Gadolinium enhancement, T2 SPACE, FLAIR 3D, DWI, PWI.

Postoperative late MRI: T1 MPRAGE +/− Gadolinium enhancement, T2 SPACE, FLAIR 3D, DWI, PWI.

### 2.3. MRI Volumetric Assessment

Tumor volume was measured with a commercially available and widely used neuro-navigation software (Elements^®^, BrainLab AG, München, Germany). Firstly, a semi-automatic image fusion was performed with the above-mentioned sequences at all time points. The image fusion enables the direct anatomical overlay of MRI images, resulting in a more precise analysis of potential FLAIR/T2 lesions. In our opinion, differentiation between residual tumor and surgically induced changes is facilitated by this approach. Furthermore, a volumetric assessment was performed based on manual segmentation of T2 and FLAIR images in preoperative, intraoperative, ultra-early, early and late postoperative MRI scans, in cooperation with the department of neuroradiology. DWI images were included in the analysis to rule out infarctions and to avoid the possibility that potential large areas were erroneously assigned to residual tumor. Hence, apart from areas likely to be an infarction, all T2 and FLAIR changes were volumetrically assessed. A differentiation of residual tumor and surgically induced changes was not performed by the reviewers during the volumetric assessment. Time between skin closure and ultra-early or early MRI was noted as well.

### 2.4. Data Analysis

The data of 16 patients were evaluated. At 3–4 months follow-up, tumor volume was evaluated. Statistical analysis was performed using SPSS 26.0 (Lead Technologies, INC, Charlotte, NC, USA). Wilcoxon and Fisher’s exact tests were used for the analysis. Correlation using Pearson’s test was calculated. The study was conducted according to the international Declaration of Helsinki. An approval from the local ethic committee was obtained.

## 3. Results

### 3.1. Patient Characteristics

A total number of 16 patients was assessed. Only patients with complete postoperative imaging including iMRI, ultra-early and early postoperative MRI were selected for further evaluation. The most common histological subtype was diffuse astrocytoma (Table 1), followed by oligodendroglioma (Table 1). The majority of patients were females (Table 1). Glioma WHO II was confirmed in 62.5% of cases (Table 1). IDH 1 or 2 mutation was found in 62.5% (Table 1). The basic characteristics are depicted in Table 1. The right hemisphere was affected in 37.5% (*n* = 6), left side in 62.5% (*n* = 10). Frontal lobe was the mostly common tumor location (62.5%, *n* = 10), followed by parietal lobe (25%, *n* = 4) and temporal lobe (6.3%, *n* = 1). Insular glioma was treated in 1 case (6.3%).

### 3.2. Volumetric and Statistical Analysis

**Figure 1:**
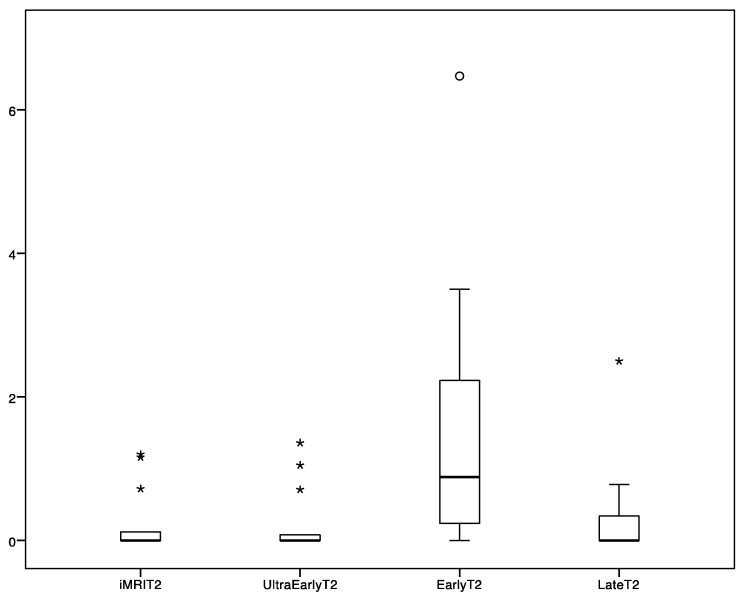
T2 tumor volumes according to different postoperative MRI scans (volume in cm^3^).

**Figure 2:**
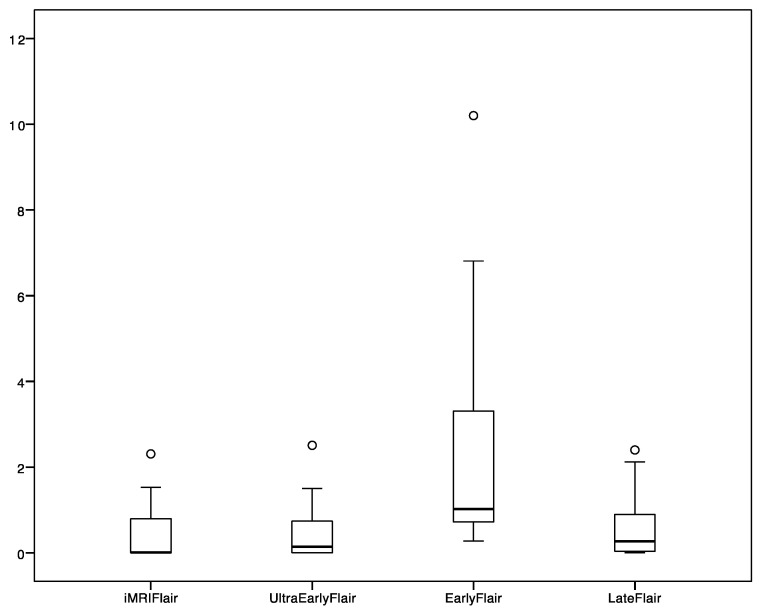
FLAIR tumor volumes according to different postoperative MRI scans (volume in cm^3^).

**Figure 3:**
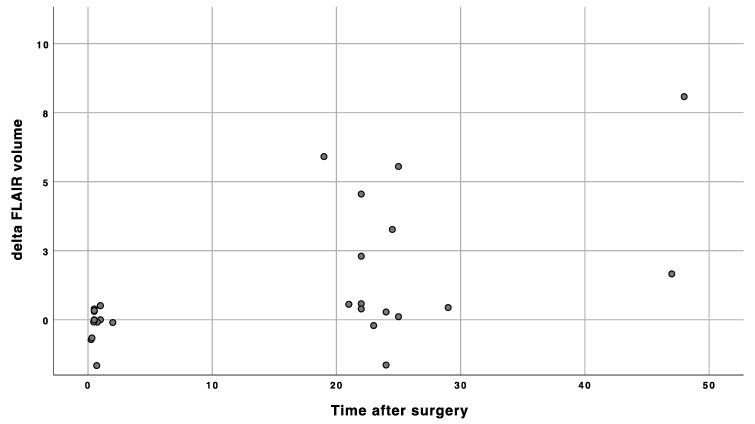
The time correlation between the difference of early or ultra-early postoperative FLAIR tumor volume and late postoperative FLAIR tumor volume (volume in cm^3^, time in hours).

Tumor volume measurements are depicted in the Table 2. Median time to ultra-early scans was 0.5 h and median time to early MRI scans was 23.5 h. We found a significant difference between FLAIR/T2 tumor volumes performed as ultra-early and early images and, simultaneously, between early and late volumes (Table 2). Furthermore, intraoperative MRI volumes without further resection depicted tumor volume similarly as ultra-early and late controls (Table 2). FLAIR/T2 tumor volumes are depicted in Figure 1 and Figure 2. We identified no significant difference between iMRI or ultra-early and late FLAIR/T2 volumes (Table 2). FLAIR/T2 tumor volumes are depicted in Figure 1 and Figure 2, respectively. According to DWI, an ischemic lesion distant from main tumor was found in 1 patient. Only FLAIR/T2 changes adjacent to main tumor were included in the analysis of this patient. The difference between early or ultra-early postoperative FLAIR tumor volume and late postoperative FLAIR tumor volume was calculated and depicted, together with the time difference between postoperative imaging and wound closure in Figure 3. Furthermore, we found a significant correlation of FLAIR tumor volumes between iMRI, ultra-early MRI and late MRI (Table 3).

We have additionally performed a subgroup analysis of IDH positive gliomas and found similarly significant differences between early and ultra-early (*p* = 0.013), as well as between early and late FLAIR volumes (*p* = 0.038). There was no significant difference between ultra-early and late FLAIR volumes. The difference between iMRI FLAIR and early FLAIR volumes also showed a significant difference (*p* = 0.038). In the evaluation of T2 sequences in IDH positive patients, we found a significant difference between ultra-early and early MRI (*p* = 0.008) and a borderline significance between iMRI and early T2 volume (*p* = 0.05). We found no significant difference between late and ultra-early and between ultra-early and iMRI T2 volume. For T2 volume in this subgroup analysis, there was no statistical difference between late and early MRI.

Furthermore, we have added the subgroup analysis of patients with MGMT positive gliomas and found similar results: FLAIR/T2 volume showed a significant difference between iMRI and early MRI (FLAIR *p* = 0.009 and T2 *p* = 0.015), between ultra-early and early MRI (FLAIR *p* = 0.013 and T2 *p* = 0.005) and between early and late MRI (FLAIR *p* = 0.007 and T2 *p* = 0.011). We found no significant differences between iMRI and ultra-early MRI, ultra-early and late MRI and between iMRI and late MRI FLAIR/T2 volumes.

Additionally, we found a significant correlation between FLAIR and T2 volumes in all perioperative MRI images (Tumor volume *p* < 0.001, *p* = 0.986, iMRT *p* < 0.001, r = 0.969, ultra-early MRI *p* < 0.001, r = 0.932, early MRI *p* = 0.001, r = 0,760 and late MRI *p* = 0.001, r = 0.783)

**Figure 4:**
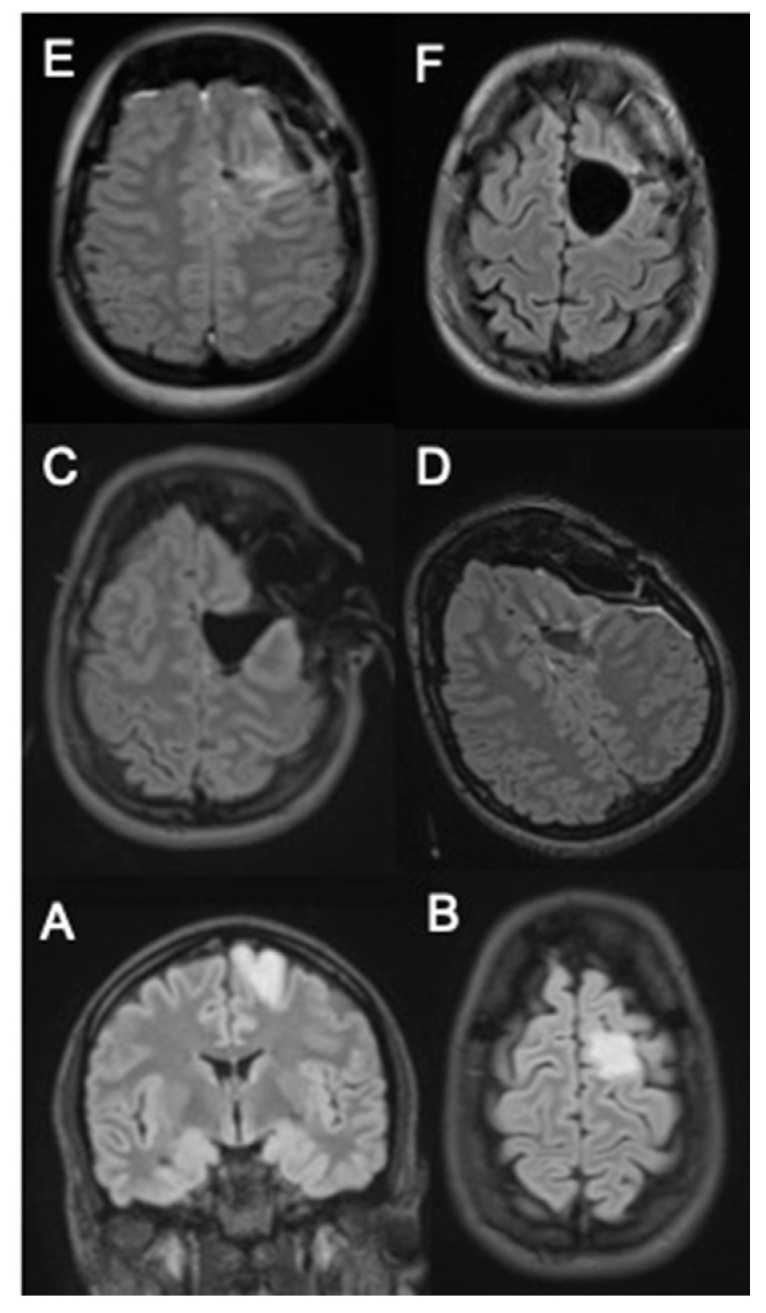
(**A**,**B**) hyperintense tumor mass without contrast enhancement on coronar and axial FLAIR. (**C**) intraoperative and (**D**) ultra-early postoperative axial MRI FLAIR images. (**E**) early postoperative axial FLAIR MRI and (**F**) late axial follow-up FLAIR MRI 3 months after surgery.

**Figure 5:**
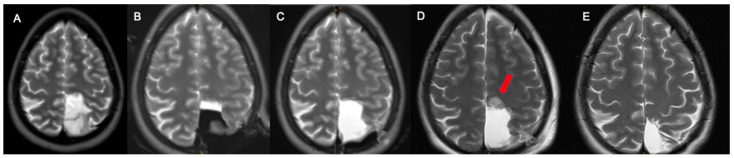
(**A**) shows T2 hyperintense lesion. (**B**) depicts intraoperative MRI and gross total resection of tumor confirmed in ultra-early MRI (**C**). (**D**) shows early postoperative MRI and surgical induced artefacts mimicking (red arrow) residual tumor. (**E**) late follow up MRI confirmed gross total resection.

Illustrative case:

A 36-year-old female patient experienced an episode of temporary visual disturbance. An MRI scan detected a left frontal mass lesion without gadolinium enhancement and with hyperintense signal abnormality in FLAIR sequences (Figure 4A,B). Due to high suspicion of low-grade glioma, iMRI-assisted surgery was performed. iMRI and ultra-early MRI after skin suture confirmed gross total resection (Figure 4C,D). An early postoperative MRI (Figure 4E) showed an increased hyperintense signal of resection cavity borders compared to iMRI and ultra-early MRI. The histopathological and molecular analysis confirmed the oligoadendroglioma WHO°II. No permanent neurological deficit was documented in follow-up 3 months after the surgery. In late postoperative MRI, FLAIR showed GTR similarly to iMRI and ultra-early MRI (Figure 4F). No surgically induced changes are visible in the late postoperative MRI. Additionally, Figure 5A–E depicts an example of another case after resection of diffuse astrocytoma. GTR was confirmed by iMRI (Figure 5B) and ultra-early MRI (Figure 5C). Early T2 showed surgically induced artefacts resembling residual tumor (Figure 5D). Late MRI confirmed GTR (Figure 5E).

## 4. Discussion

The treatment and postoperative management of patients with LGG is challenging due to its infiltrative growth, which results in inevitable recurrence [10,11]. Additional intraoperative imaging techniques were confirmed as important tools in increasing the extent of resection (EoR) and the number of gross total resections (GTR) in LGG. Both techniques are highly beneficial in increasing PFS and OS [12,13,14,15]. However, the evaluation of early postoperative FLAIR/T2 (<48 h) signal alterations, and the identification of real tumor remnants on early postoperative images can be misleading as a result of surgically induced changes. FLAIR seems to especially overestimate tumor borders on these images [7]. Delineation of tumor remnants in LGG are based on FLAIR and T2 sequences, which play an important role in patient follow-up and in further therapy management of LGG. The timing of adjuvant treatment and its potential impact on further survival has been the subject of many discussions [1,16,17]. Based on an RTOG 9802 study, patients with tumor remnants are assigned for further adjuvant treatment by many neuro-oncologists. Additionally to the side effects of a likely unnecessary treatment, a potential negative influence of early alkylating treatment in LGG has been reported [6,18,19,20].

The use of iMRI after final tumor resection, or an MRI directly after skin closure, might delineate tumor borders more precisely [8]. In order to confirm this hypothesis, we have evaluated the standardized prospective collected MRI data from the Log-Glio registry and confirmed that an ultra-early MRI directly after surgery, or an intraoperative MRI without additional resection, may be superior compared to standard postoperative imaging at 24–48 h for the identification of potential tumor remnants and distinguishing these from surgically induced artifacts.

The multicenter retrospective analysis of patients treated with IDH-positive LGGs showed an inferior PFS and OS to patients who underwent adjuvant treatment directly after surgery [18]. Additionally, even the subgroup analysis of so called high-risk patients, older than 40 years and/or harboring a tumor remnant, showed a significantly improved survival without adjuvant treatment [18]. Based on these results, the critical identification of tumor remnants on postoperative MRI images has a great importance.

According to our results, an ultra-early MRI directly after skin closure depicts fewer surgically induced artifacts on FLAIR/T2 images as an early MRI after surgery from 20 to 48 h. Similarly, intraoperative FLAIR and T2 without further tumor resection also delineates tumor remnants more precisely than an early MRI. The difference between intraoperative and ultra-early FLAIR/T2 abnormalities on MRI showed no significant difference. Despite this, an intraoperative shift and loss of CSF iMRI seems to depict tumor remnants without contrast enhancement more precisely than an early postoperative MRI. The advantage of presented data is the standardized prospective protocol, including preoperative, intraoperative, ultra-early and early, as well as late MRI, resulting in a homogeneous cohort which allows for a more accurate volumetric evaluation.

The direct evaluation of GTR after surgery has further important implications on the prognosis and survival of patients treated with LGG [21,22,23]. An early evaluation of GTR is relevant in regard to surgical quality control, especially when discussing surgical results with the respective patient. Further, to specifically distinguish between residual tumor and surgically induced changes facilitates the decision for an early re-resection if there is a resectable tumor remnant.

Considerable tumor rest in LGGs is a potential source of an early malignisation [22] and it has been proposed that tumor remnant after initial surgery is the major predictor for tumor recurrence [24]. Furthermore, even small tumor remnants of a diffuse glioma have been shown to have a negative impact on OS, which suggest a need for early second-look surgery [25]. Consequently, a routine MRI scan up to 48 h after surgery might become obsolete, since it overestimates the true extent of the disease.

The delineation of LGG remnants is an important factor for later follow-up, since LGG presents itself slowly, and is a disease resulting inevitably in recurrence [2,26]. Therefore, a more precise definition of tumor growth velocity, according to tumor remnants in ultra-early and follow up MRI scans, might be beneficial for further treatment and therapy planning, as well as a better estimation of further prognosis. Additionally, molecular characteristics became a crucial player in the glioma therapy and prognosis prediction. IDH wildtype non-enhancing gliomas request further adjuvant treatment since they are treated as high-grade gliomas [27,28]. Based on that, the delineation of true tumor remnants and their evaluation in regard to tumor progress in follow-up scans has even more relevance for the monitoring of therapy success.

Ischemia as a consequence of tumor resection plays an important role in inducing T2/FLAIR changes and contribute to the overestimation of tumor remnants. Different approaches, such as the probabilistic segmentation of ADC maps, were suggested in the literature [29]. We have included DWI images in the volumetric analysis of intraoperative and postoperative changes in order to exclude large ischemic changes.

A limitation of our study is a relatively small number of patients, even if it incorporated the standardized prospective protocols. MRI imaging was performed on different scanners for intraoperative, early postoperative and late postoperative MRI. The difference between 1.5 and 3 T might be a potential bias of this study. The manual segmentation is always a source of potential bias, which we tried to address by using two independent reviewers. Our cohort is inhomogeneous in regard to the final WHO° classification including gliomas graded as III and IV. However, we have only evaluated tumors without contrast enhancement, so that the evaluation of MRI imaging was similar to gliomas WHO II. Additionally, we performed the subgroup analysis of IDH-mutated patients and found similar results for FLAIR and T2, with the exception of a comparison between early and late T2 volume. Moreover, we found similar differences in the subgroup analysis of patients with a methylated MGMT promotor.

Sending a patient to an ultra-early MRI directly after surgery results in some logistical difficulties, such as the availability of MRI or compliance of patients. Post-anesthesia recovery and the difficulty of neurological monitoring during MRI scanning may result in imaging delays. Furthermore, it is an additional stress factor after surgery for patients. Our data are limited since they do not provide volumetric data on additional time points between 2 and 20 h after surgery, which was mainly based on logistical clinical constrains, as mentioned above. We would expect a linear increase in surgically induced artifacts during that time. However, further data are needed to assess whether the optimal “ultra-early” time period could also be stretched to several hours after surgery. Based on our experienced with long-lasting LGG surgeries with several iMRI scans, this is likely possible. Hence, each hospital has to find its optimal timing for a postoperative MRI.

Our data support imaging as soon as possible after surgery to avoid increasing imaging artifacts. An ultra-early MRI in LGGs might become a relevant diagnostic step, even in centers without an intraoperative MRI scanner, which could improve the evaluation of residual tumor. Furthermore, in centers using iMRI, ultra-early or early MRI scanning after surgery seem to be redundant, if no additional resection was performed after iMRI.

## 5. Conclusions

Postoperative MRI performed from 20 to 48 h after surgery overestimates tumor borders and might lead to false positive findings. A false stratification in high-risk patients might result in the application of adjuvant treatment immediately after surgery. An ultra-early postoperative MRI performed within one hour after skin closure might be more appropriate for the delineation of tumor remnants in gliomas without contrast enhancement and might result in better treatment evaluation, patient consultation and even potential feedback for the surgeon. An intraoperative MRI without additional resection and with an open skull seems to be a suitable alternative to an ultra-early MRI scan, and is less prone to surgically induced artifacts than early postoperative MRI.

## Figures and Tables

**Table 1:** Tumor and patients’ characteristics.

	Total
*n*	16
Eloquent location	68.8% (11)
female ratio	56.3% (9)
Astrocytoma	43.8% (7)
Oligodendroglioma	37.5% (6)
Others	18.8% (3)
IDH mutation	62.5% (10)
MGMT methylated	68.8% (11)
WHO°I	12.5% (2)
WHO°II	62.5% (10)
WHO°III	18.8% (3)
WHO°IV	6.3% (1)

**Table 2:** Differences between intraoperative T2 and Flair changes (median volume, cm^3^).

T2 Volume (11.1)	Intraoperative MRI (<0.001)	Ultra-Early MRI (<0.001)	Early MRI (0.94)	Late Follow Up MRI (<0.001)
Intraoperative MRI (0.2)		*p* = 0.499	***p* = 0.035**	*p* = 0.674
Ultra-early MRI (<0.001)	*p* = 0.499		***p* = 0.003**	*p* = 0.575
Early MRI (1.5)	***p* = 0.035**	***p* = 0.003**		***p* = 0.019**
Late follow up MRI (0.05)	*p* = 0.674	*p* = 0.575	***p* = 0.019**	

**Table 3:** Correlation between FLAIR tumor volumes in different postoperative MRI scans.

Pearson Correlation (FLAIR)	Intraoperative MRI	Ultra-Early MRI	Early MRI	Late Follow Up MRI
Intraoperative MRI		***p* < 0.001**	*p* = 0.658	***p* < 0.001**
		**R = 0.828**	R = 0.125	**R = 0.882**
Ultra-early MRI	***p* < 0.001**		*p* = 0.116	***p* = 0.002**
	**R = 0.828**		R = 0.408	**R = 0.740**
Early MRI	*p* = 0.658	*p* = 0.116		*p* = 0.256
	R = 0.125	R = 0.408		R = 0.313
Late follow up MRI	***p* < 0.001**	***p* = 0.002**	*p* = 0.256	
	**R = 0.882**	**R = 0.740**	R = 0.313	

## Data Availability

Data available on request.
